# Human parietal epithelial cells (PECs) and proteinuria in lupus nephritis: a role for ClC-5, megalin, and cubilin?

**DOI:** 10.1007/s40620-023-01725-6

**Published:** 2023-08-18

**Authors:** Monica Ceol, Lisa Gianesello, Hernan Trimarchi, Alberto Migliorini, Giovanna Priante, Claudia M. Radu, Elena Naso, Annalisa Angelini, Lorenzo A. Calò, Franca Anglani, Dorella Del Prete

**Affiliations:** 1 Nephrology Unit- Kidney Histomorphology and Molecular Biology Laboratory, Department of Medicine-DIMED, University of Padua, Via Giustiniani, 2, 235128 Padua, Italy; 2 Nephrology Service, Hospital Británico de Buenos Aires, Buenos Aires, Argentina; 3 General Internal Medicine and Thrombotic and Hemorrhagic Diseases Unit, Department of Medicine, University of Padua, Padua, Italy; 4 Cardiovascular Pathology and Pathological Anatomy, Department of Cardiac, Thoracic, Vascular Sciences and Public Health, University of Padua, Padua, Italy

**Keywords:** ClC-5, Megalin, Cubilin, Hypertrophic PECs, Kidney biopsy

## Abstract

**Background:**

Parietal epithelial cells are a heterogeneous population of cells located on Bowman’s capsule. These cells are known to internalize albumin with a still undetermined mechanism, although albumin has been shown to induce phenotypic changes in parietal epithelial cells. Proximal tubular cells are the main actors in albumin handling via the macromolecular complex composed by ClC-5, megalin, and cubilin. This study investigated the role of ClC-5, megalin, and cubilin in the parietal epithelial cells of kidney biopsies from proteinuric lupus nephritis patients and control subjects and identified phenotypical changes occurring in the pathological milieu.

**Methods:**

Immunohistochemistry and immunofluorescence analyses for ClC-5, megalin, cubilin, ANXA3, podocalyxin, CD24, CD44, HSA, and LTA marker were performed on 23 kidney biopsies from patients with Lupus Nephritis and 9 control biopsies (obtained from nephrectomies for renal cancer).

**Results:**

Two sub-populations of hypertrophic parietal epithelial cells ANXA3^+^/Podocalyxin^−^/CD44^−^, both expressing ClC-5, megalin, and cubilin and located at the tubular pole, were identified and characterized: the first one, CD24^+^/HSA^−^/LTA^−^ had characteristics of human adult parietal epithelial multipotent progenitors, the second one, CD24^−^/LTA^+^/HSA^+^ committed to become phenotypically proximal tubular cells. The number of glomeruli presenting hypertrophic parietal epithelial cells positive for ClC-5, megalin, and cubilin were significantly higher in lupus nephritis patients than in controls.

**Conclusions:**

Our results may provide further insight into the role of hypertrophic parietal epithelial cells located at the tubular pole and their possible involvement in protein endocytosis in lupus nephritis patients. These data also suggest that the presence of hypertrophic parietal epithelial cells in Bowman's capsule represents a potential resource for responding to protein overload observed in other glomerulonephritis.

**Graphical abstract:**

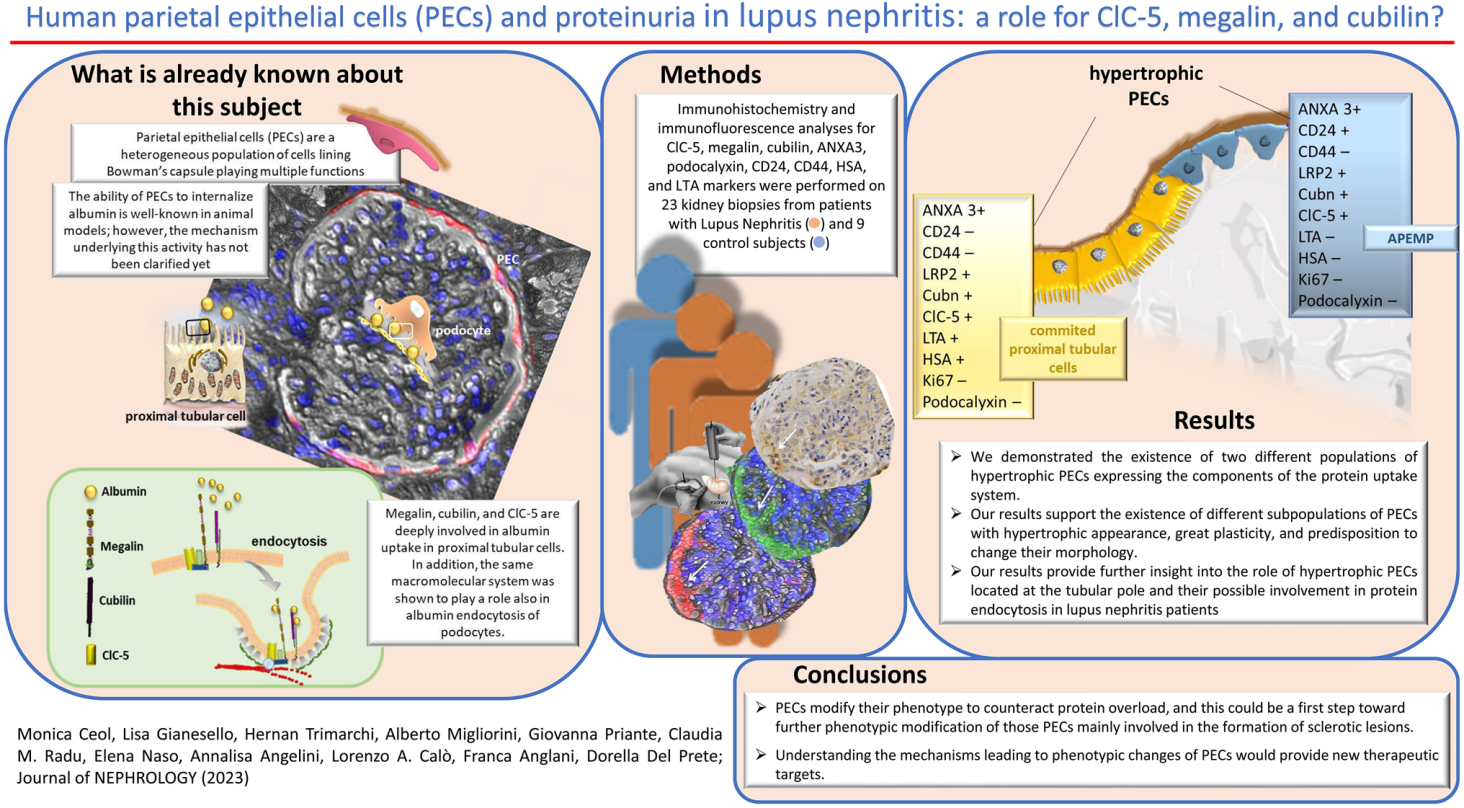

**Supplementary Information:**

The online version contains supplementary material available at 10.1007/s40620-023-01725-6.

## Introduction

In 2009, Ronconi et al. demonstrated the existence of human renal progenitors located in Bowman’s capsule [1]. This is a strategic location due to its close proximity to both tubular cells and podocytes. Many studies focused on the pathogenesis of hyperplastic lesions and the formation of crescents in glomerulopathies, providing evidence that renal progenitor cells located in Bowman’s capsule may differentiate into podocytes under certain clinical circumstances [2, 3].

Parietal epithelial cells (PECs) are a heterogeneous cell population lining the outer membrane of Bowman’s capsule characterized by specific molecular markers. Besides the classical flat PECs, a subpopulation of cuboidal (cPECs) and intermediate PECs (iPECs) has also been identified [4]. Mature PECs are recognizable by the expression of annexin A3 (ANXA3), LKIV69, Pax-2, Pax-8, and claudin-1. Podocyte-committed progenitors are detectable by the co-expression of the podocyte marker podocalyxin and the progenitor markers CD24 and CD133/2. Adult parietal epithelial pluripotent progenitors (APEMP) have been shown to express CD24 and CD133/2 but not podocyte or tubular markers [5]. Activated PECs play a key role in the pathogenesis of glomerulosclerosis and possess the capacity to proliferate, migrate and produce extracellular matrix. These cells are known to express the CD44 marker [2, 3].

The cellular protein uptake machinery composed of megalin, cubilin, and ClC-5 is normally present in proximal tubular cells (PTCs) but it is also present in human podocytes [6–8]. It was also reported that mouse and rat PECs expressing megalin and cubilin were able to internalize albumin in normal and overload conditions [9, 10].

Lupus nephritis (LN) is a frequent complication of systemic lupus erythematosus, a systemic autoimmune disease [11] that has a major impact on the prognosis of the disease, resulting in a risk of end-stage renal disease. The pathogenesis of LN involves genetic, epigenetic, immunoregulatory, hormonal, and environmental phenomena. Lupus nephritis is associated with a wide spectrum of kidney lesions, mostly characterized by glomerular involvement. Mesangial cells and podocytes have been identified as direct targets of immune complexes through specific epitopes [12]. Proteinuria is an important marker of LN. Crosstalk between podocytes and PECs in some proliferative glomerular diseases is well known [3], while the role of PECs in the mechanism of protein uptake needs to be further explored.

In this study we aimed to investigate the role of ClC-5, megalin, and cubilin in PECs of kidney biopsies from patients with lupus nephritis as compared to control kidneys, and through the characterization of the different PEC subpopulations, to identify phenotypical changes of PECs occurring in the pathological milieu.

## Materials and methods

### Patients

Serial sections of 23 kidney biopsies from patients with LN, were performed for diagnostic purposes and were available for immunolabeling studies. Nine control kidney biopsies containing cortex areas (C) obtained from nephrectomies for renal cancer (sites remote from the tumor-bearing renal tissue), showing normal morphology and negative immunofluorescence (IF), were also analyzed. Patients’ clinical details are reported in Table 1. The study was approved by Padua University Hospital’s Ethical Committee, protocol 0007452 (February 1st, 2018) and all patients gave informed consent.

**Table 1:** Patients’ clinical and histological details

Patients	Control (*n* = 9)	LN (*n* = 23)
Age (years)	55 (46–71)	34 (16–45)
Gender	7 M	5 M
uProt g/day	na	3.63 (0.23–10)
sCreat mg/dl	1.13 (0.93–1.44)	0.71 (0.35–1.2)
ISN/RPS	–	3 pts stage II 3 pts stage III 15 pts stage IV 2 pts stage V
Total number of glomeruli	82	158
Number of sclerotic glomeruli	0/82	6/158
Number of glomeruli with hypertrophic PECs	2/82	63/158
Number of glomeruli with crescents	0/82	6/158

*uProt* urinary protein excretion, *sCreat* serum creatinine

### Immunohistochemistry

Immunohistochemistry (IHC) was conducted on formalin-fixed, paraffin-embedded sections using an indirect immunoperoxidase method. Specimens were treated as previously described [8] and incubated overnight at 4 °C in a humidified chamber with rabbit anti-human ClC-5 antibody diluted in PBS (Table 2). Immunolabeling specificity was confirmed by incubating without any primary antibody. Images were acquired with the Diaplan light microscope (Leitz, Como, Italy) and a 20X/0.45 lens using a Micropublisher 5.0 RTV camera (Teledyne QImaging, Surrey, BC, Canada).

**Table 2:** Semiquantitative analysis of glomeruli with PECs positive for ClC-5, megalin, and cubilin

	Control (*n* = 9) (%)	LN (*n* = 23) (%)
ClC-5	25	25
Megalin	27	17
Cubilin	15.5	11.5

Number of glomeruli with positive PECs (flat and hypertrophic) vs number of total glomeruli expressed in percentage

### Immunofluorescence

Immunofluorescence analyses were performed on serial paraffin embedded sections of kidney biopsies. Samples were treated as previously described [7] and incubated overnight at 4 °C in a humidified chamber with specific primary antibodies diluted in PBS 5% BSA (Supplementary table 1). Specific fluorescent secondary antibodies were appropriately diluted in PBS 5% BSA (Supplementary table 1). Nuclei were counterstained with DAPI (4′,6-diamidino-2-phenylindole) (Vector Laboratories Burlingame, CA) and kept at 4 °C in darkness to dry. Negative controls were run by omitting primary antibodies.

### LTA staining

Fluorescein-labeled Lotus Tetragonolobus Lectin (LTA, LTL-FITC; Vector Laboratories, Burlingame, CA) staining was performed by incubating serial kidney sections with FITC-LTA diluted 1:100 in PBS 5% BSA for 1 h at room temperature. Nuclei were counterstained with DAPI and kept at 4 °C in darkness to dry.

### Signal acquisition

All sections were stained and analyzed simultaneously for the same antibody to exclude artifacts due to variable decay of the fluorochromes. Images were acquired with a DMI6000CS fluorescence microscope (Leica Microsystems, Milan, Italy) with a 40X/0.6 and a 63X/1.4 lens using a DFC365FX camera (Leica Microsystems), and analyzed by the LAS-AF software 3.1.1 (Leica Microsystems).

All kidney samples from controls and proteinuric patients were immunostained for ANXA3, megalin, cubilin, ClC-5, and human serum albumin (HSA). Serial sections of selected patients (*n* = 13) showing PECs with a hypertrophic appearance were characterized for the expression of CD24, CD44, Podocalyxin, LTA, and Ki67.

### Semiquantitative analysis

Parietal epithelial cells were identified on the basis of morphological features, capsular localization, and ANXA3 positivity. Glomeruli in which a boundary between the glomerular flocculus and Bowman’s capsule could not be clearly defined and those with global sclerosis were excluded. We reduced sampling bias and artifacts due to sample orientation as much as possible by analyzing a sufficient number of glomeruli. Semiquantitative analysis was performed counting the number of glomeruli with positive PECs for each marker in C and LN biopsies.

### Statistical analysis

Confocal analysis of co-localization rate between megalin and CD24 and between megalin and LTA was performed using the LAS-AF software (Leica Microsystems) using Pearson’s correlation coefficient (Rr). An Rr value > 0.7 was considered to indicate a strong correlation.

The Chi square test was used to compare frequencies using the R program, version 4.0.2 software [13]. A *p* value < 0.05 was considered significant.

## Results

Histological analysis and clinical parameters of the LN group are reported in Table 1. Most of the patients (*n* = 15) had stage IV according to the ISN/RPS classification, 3 patients had stage II, 3 patients had stage III, and 2 patients had stage V. In LN renal biopsies we evaluated 158 glomeruli, 3.8% were sclerotic and another 3.8% showed cellular crescents and belonged to patients in stage IV (Table 1).

Inflammatory infiltrate was present in 5 patients out of 23: one patient belonged to class II, one to class III, and three to class IV. These small numbers did not allow us to verify a possible link between inflammation and the presence of hypertrophic PECs positive for ClC-5, megalin, and cubilin.

We did, however, observe, for the first time in both C and LN glomeruli, PECs expressing the protein uptake macromolecular machinery composed of ClC-5, megalin, and cubilin which were scattered around the glomerulus and did not significantly differ between C and LN. Semiquantitative analysis considering the number of glomeruli with all positive human PECs (flat and hypertrophic) vs number of total glomeruli in C and LN are reported in Table 2.

**Fig. 1:**
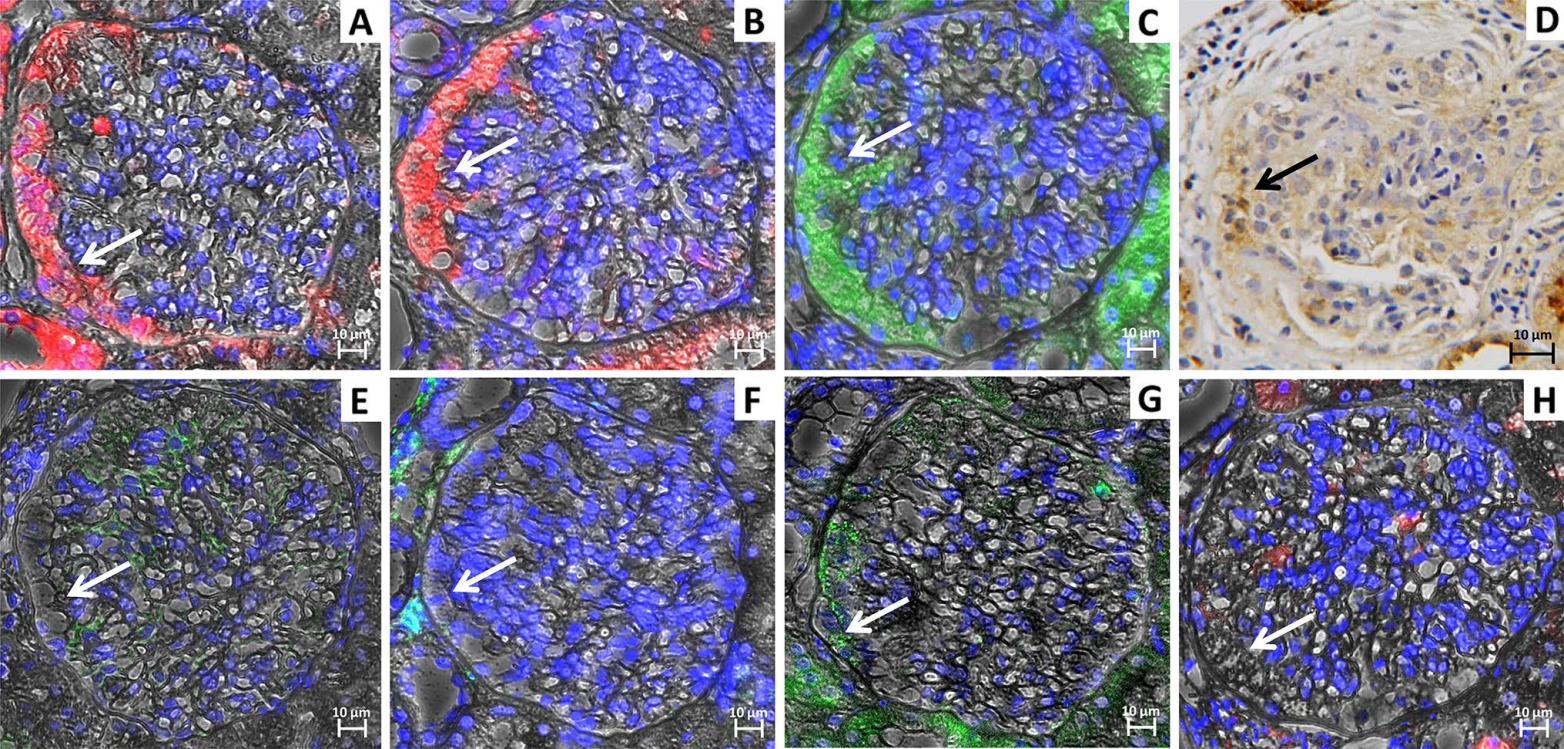
Phenotypic characterization of PECs in kidney biopsies from LN patients: identification of the CD24^+^ subpopulation expressing the protein uptake machinery. Representative images of serial sections of glomerulus with hypertrophic PECs identified by arrows. PECs **A** ANXA3^+^ (red: CFL647) showing positivity also for **B** megalin (red: CFL647), **C** cubilin (green: fluorescein isothiocyanate [FITC], **D** ClC-5 (brown: DAB), **G** CD24 (green: Alexa Fluor 488). Representative immunofluorescence images showing PECs negative for **E** podocalyxin (green: Alexa Fluor 488), **F** CD44 (green: Alexa Fluor 488) and **H** HSA (red: CFL647). Nuclei were counterstained with DAPI (IF images blue), and Mayer hematoxylin (IHC image blue). Immunofluorescence magnification 40X/0.6, IHC magnification 20X/0.45. Scale bar 10 µm

**Fig. 2:**
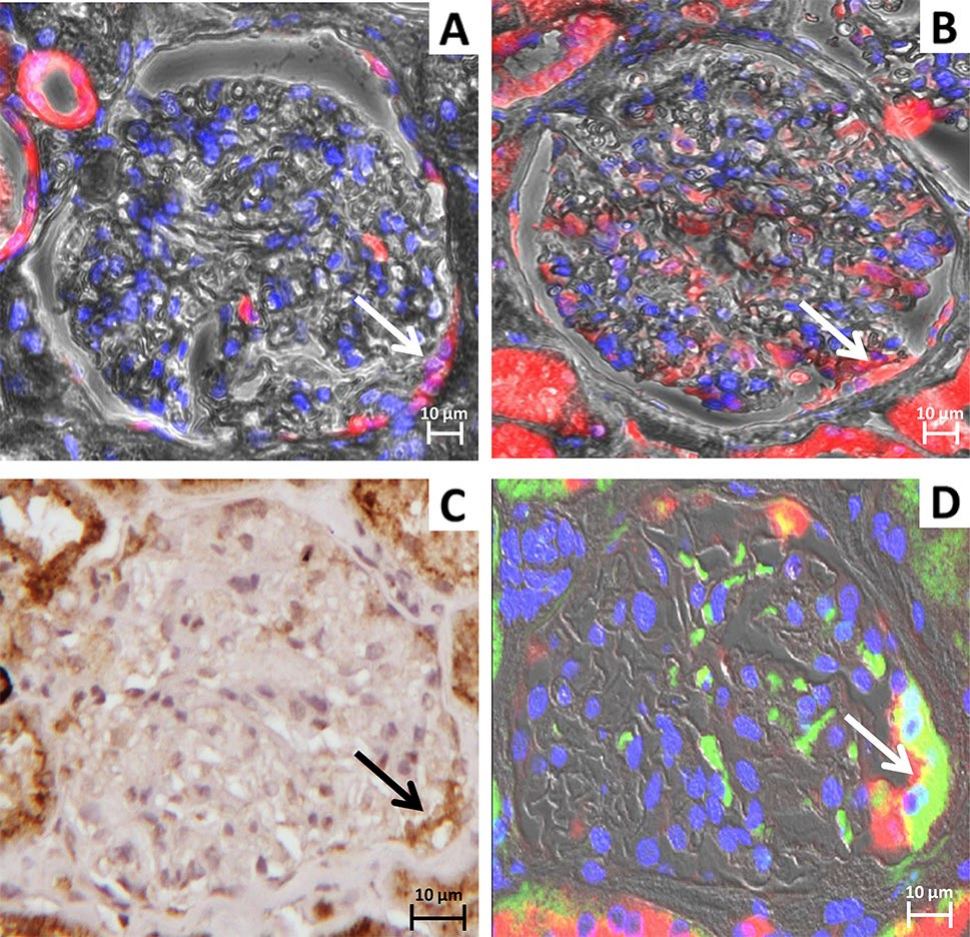
Phenotypic characterization of PECs in control kidney biopsies: identification of the CD24^+^ subpopulation expressing the protein uptake machinery. Representative images of serial sections of glomerulus with hypertrophic PECs identified by arrows. Immunofluorescence microscopy demonstrating positivity for **A** ANXA3^+^ (red: CFL647) and **B** megalin (red: CFL647), magnification 40X/0.6. **C** Immunohistochemistry staining showing positive signal for ClC-5 (brown: DAB), magnification 20X/0.45. **D** Confocal image showing co-localization between megalin (red: CFL647) and CD24 (green: Alexa Fluor 488) in yellow/orange PECs, magnification 63X/1.4. Nuclei were counterstained with DAPI (IF images blue), and Mayer hematoxylin (IHC image blue). Scale bar 10 µm

Different morphologies of ANXA3 positive cells were observed. Besides the classical flat PECs, we noticed in both control and proteinuric biopsies some cells showing a hypertrophic appearance with larger size and more represented cytoplasm than flat PECs mainly located at the tubular pole of the glomerulus (Figs. 1A, 2A). Hypertrophic PECs displayed positivity for megalin, cubilin, and ClC-5. Table 3 shows the percentage of hypertrophic PECs expressing the components of the macromolecular system in C and in biopsies of LN patients. The different expression between the number of glomeruli with positive hypertrophic PECs in C and LN was statistically different for all molecules (ClC-5 *χ*^2^ 8.17 *p* = 0.0042; megalin *χ*^2^ 17.4537 *p* = 0.0000; cubilin *χ*^2^ 15.3634 *p* = 0.00002). These proteins were not always co-expressed by the same hypertrophic cells (Figs. 1B–D, 2B , C). In order to better characterize which type of PEC was able to express the components of the tubular uptake machinery, we tested different markers: CD24 for progenitor cells, CD44 for pro-sclerotic activation, podocalyxin for podocytes, LTA for PTC brush border, and Ki67 for proliferating cells. Furthermore, anti-HSA was used to detect albumin. Semiquantitative analysis performed on CD24 and CD44 positive PECs revealed, though only for CD24, a significant difference between C and LN (*χ*^2^ 16.5968 *p* = 0.00004).

**Table 3:** Semiquantitative analysis of glomeruli with hypertrophic PECs positive for ClC-5, megalin, and cubilin

	Control (*n* = 9) (%)	LN (*n* = 23) (%)
ClC-5	2.4	14
Megalin	1.6	13
Cubilin	0.5	9.2

Number of glomeruli with hypertrophic positive PECs vs number of total glomeruli expressed in percentage

Hypertrophic PECs positive for megalin, cubilin, and ClC-5 at the tubular pole were negative for podocalyxin, CD44 (Fig. 1E and F), and HSA (Fig. 1H), with scattered positivity for the marker CD24 both in LN and in C biopsies (Figs. 1G and 2D). The degree of co-localization between megalin and CD24 signals was highly correlated both in LN and C biopsies (Rr 0.81 ± 0.07). Ki67 and LTA signals were absent (data not shown).

**Fig. 3:**
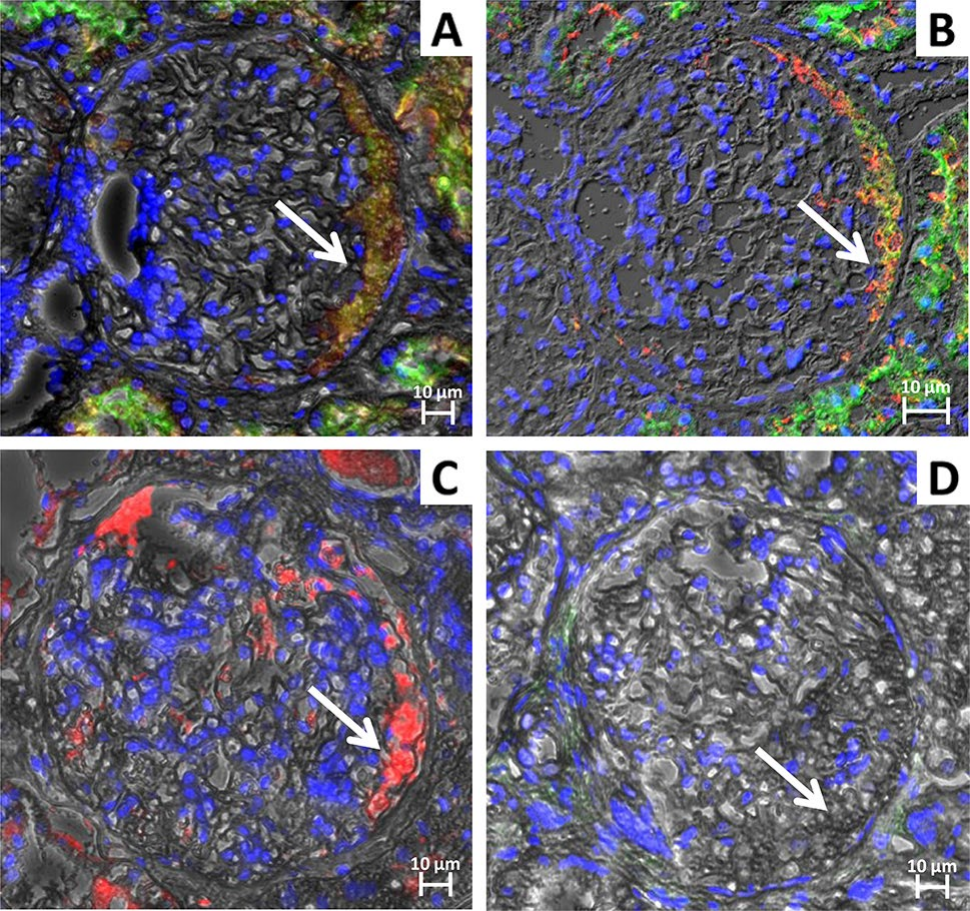
Phenotypic characterization of PECs in kidney biopsies from proteinuric patients: identification of a subpopulation of CD24^−^ PECs committed to PTC phenotype expressing the protein uptake machinery. Representative images of serial sections of glomerulus with hypertrophic PECs identified by arrows. **A** Immunofluorescence image showing co-localization between megalin (red: CFL647) and cubilin (green: FITC) in yellow/orange PECs, magnification 40X/0.6. **B** Confocal image showing co-localization between megalin (red: CFL647) and LTA (green: FITC) in yellow/orange PECs, magnification 63X/1.4. **C** Immunofluorescence microscopy showing the presence of HSA (red: CFL647) and **D** absence of CD24 signal (green: Alexa Fluor 488) in the same PECs, magnification 40X/0.6. Nuclei were counterstained with DAPI (Blue). Scale bar 10 µm

On the other hand, we observed that there was a distinct population of hypertrophic PECs showing co-localization for megalin, cubilin, and LTA (Rr 0.82 ± 0.06) (Fig. 3A and B). These cells were also positive for HSA (Fig. 3C) and negative for CD24 (Fig. 3D). When analyzing the regions with these hypertrophic PECs we observed that the HSA and LTA signals were scattered in a distributed manner. The two subpopulations of hypertrophic PECs described in our study are reported in Supplementary Fig. 1.

## Discussion

Recently, PECs have attracted a great deal of attention in an attempt to clarify their molecular phenotype and understand their function and responses in normal and in pathological conditions [4, 14].

The ability of PECs to internalize albumin is well-known in animal models [9]. However, the mechanism underlying this activity is yet to be clarified.

ClC-5, megalin, and cubilin are involved in protein uptake, and for the first time our study identified their presence also in the PECs of patients with lupus nephropathy and proteinura.

In humans, CD24 and CD133 are used to characterize cells with stem or progenitor properties [1]. The co-expression of CD24 and CD133 has been identified in a subset of PECs on the Bowman’s capsule of adult human kidneys [5]. We identified a CD24^+^/Podocalyxin^−^ subpopulation of PECs with hypertrophic appearance, expressing the protein uptake system components that we speculate to be APEMP, supporting the recently proposed hypothesis of the existence of tubular committed progenitor cells among PECs [3, 4].

The cells we identified could morphologically be iPECs sharing similarities with the tubular cell phenotype, without having reached the functional differentiation state, as suggested by the absence of the apical brush border.

We also demonstrated the presence of another population of CD24^−^/LTA^+^ PECs with the morphological appearance of cPECs at the tubular pole as well. They expressed ClC-5, megalin and cubilin with the ability to internalize albumin, suggesting a further step of commitment of these cells toward PTCs.

The different CD24 and LTA expression observed among hypertrophic PECs could likely be attributable to the different stages of PEC differentiation occurring between intermediate and cuboid PECs.

Many studies focused on the role of aPECs (CD44^+^/Ki67^+^) in the genesis of sclerotic lesions [3, 14], while less information is available regarding the role of PECs in the mechanism of protein uptake in normal kidney and in proteinuric nephropathies, a key step that may eventually lead to glomerulosclerosis.

The observation in our study of inactivated and non-proliferating hypertrophic PECs (CD44^−^/Ki67^−^) suggests that these cells are not involved in the pathogenesis of sclerotic lesions, at least at this stage of the nephropathy [10, 15].

The presence of hypertrophic CD24^+^ PECs, in a significantly greater number in lupus nephropathy biopsies than in controls, suggests that both proliferative glomerulonephritis and proteinuric milieu might induce an increment in these cells.

The observation that, even in controls, different subpopulations of PECs were present within the same glomerulus (with/without hypertrophy) highlights the great plasticity of PECs and suggests that these cells may change their morphology and protein expression in response to different stimuli (ischemia, proteinuria, hyperglycemia) [4].

In this study we have shown that two main sub-populations of hypertrophic PECs are present in human glomeruli, both of which express ClC-5, megalin, and cubilin. One subtype seems to show characteristics of APEMP, while the other is suggestive of the PTC phenotype.

The main limitation of our study is that we analyzed only one type of glomerular disease, lupus nephritis, both proliferative (Classes II, III and IV) and non-proliferative (Class V). Although the urinary hallmark is proteinuria, the pathophysiological pathways may differ. In this regard, we believe it would be interesting to evaluate glomerulonephritis of different etiologies, such as IgA and membranous nephropathies, to support the hypothesis that protein milieu can induce the increase in APEMPs and to demonstrate whether the phenotypic changes in PECs, described in our research, are independent or not of the immunologic pathogenesis of the underlying nephropathy.

However, our results obtained in LN biopsies may provide a starting point for further studies. Another limitation is that we did not perform characterization for progenitor cells using CD133/2, a well-known CD24 molecular partner. However, many studies report that CD133/2 and CD24 are co-expressed in the adult kidney [1, 5, 16, 17] allowing us to suppose that positivity for CD24 could identify progenitor-like cells.

In conclusion, our results describe two main sub-populations of hypertrophic PECs in human glomeruli in proteinuric proliferative and non-proliferative human lupus kidney biopsies, both of which express megalin, cubilin, and ClC-5, components of the macromolecular complex normally expressed by PTCs. The first subset seems to show characteristics of APEMP, the second is more in keeping with a PTC phenotype. In addition, our data suggest that the presence of hypertrophic PECs in the Bowman’s capsule could reflect a response to protein overload in LN, regardless of the proliferative setting. The link between proteinuria and renal disease progression remains very strong. The observation that PECs can modify their phenotype in order to counteract protein overload could be a first step toward identifying PEC phenotypes mainly involved in the formation of glomerulosclerotic lesions.

### Supplementary Information

Below is the link to the electronic supplementary material.

Supplementary file1 (DOCX 821 kb)
