# Supplementary material for: Human parietal epithelial cells (PECs) and proteinuria in lupus nephritis: a role for ClC-5, megalin, and cubilin?
Source: J Nephrol. 2023 Aug 18;36(9):2499–506. doi: 10.1007/s40620-023-01725-6 (PMC10703968; doi:10.1007/s40620-023-01725-6)
Supplement: Supplementary file 1 — Supplementary file1 (DOCX 821 kb) [file 40620_2023_1725_MOESM1_ESM.docx]

**Supplementary table 1. Antibodies used in immunohistochemistry and immunofluorescence.**

| **Target** | **Clone** | **Host** | **Manufacture** | **Code** | **Conjugation** | **Dilution** |
| --- | --- | --- | --- | --- | --- | --- |
| ClC-5 |  | rabbit | Atlas Antibody | HPA000401 |  | 1:200 |
| ANXA3 |  | rabbit | Atlas Antibody | HPA013398 |  | 1:500 |
| megalin |  | rabbit | Atlas Antibody | HPA005980 |  | 1:100 |
| cubilin |  | sheep | R&D Systems | AF 3700 |  | 1:100 |
| CD24 | SN3 | mouse | Santa Cruz Biotechnology | Sc-19585 |  | 1:20 |
| CD44 | HI44a | mouse | Immunostep | 44PU2-O1MG |  | 1:20 |
| HSA |  | rabbit | ThermoFischer Scientific | PA1-26462 |  | 1:300 |
| Podocalixin | 4F10 | mouse | Santa Cruz Biotechnology | Sc-23903 |  | 1:100 |
| Ki67 |  | rabbit | GeneTex | GTX20833 |  | 1:50 |
| Anti-mouse |  | goat | ThermoFischer Scientific | A-11001 | Alexa Fluor 488 | 1:1000 |
| Anti-sheep |  | donkey | Santa Cruz Biotechnology | Sc 2476 | FITC | 1:100 |
| Anti-rabbit |  | donkey | Santa Cruz Biotechnology | Sc 362291 | CFL 647 | 1:100 |
| Anti-rabbit |  |  | ENZO | ENZ-ACC103 | Polview Plus  HRP reagent | Ready-to-  use |

**Supplementary Figure S1. Representative image of the two hypertrophic PECs subpopulations identified in control and proteinuric kidney biopsies.**


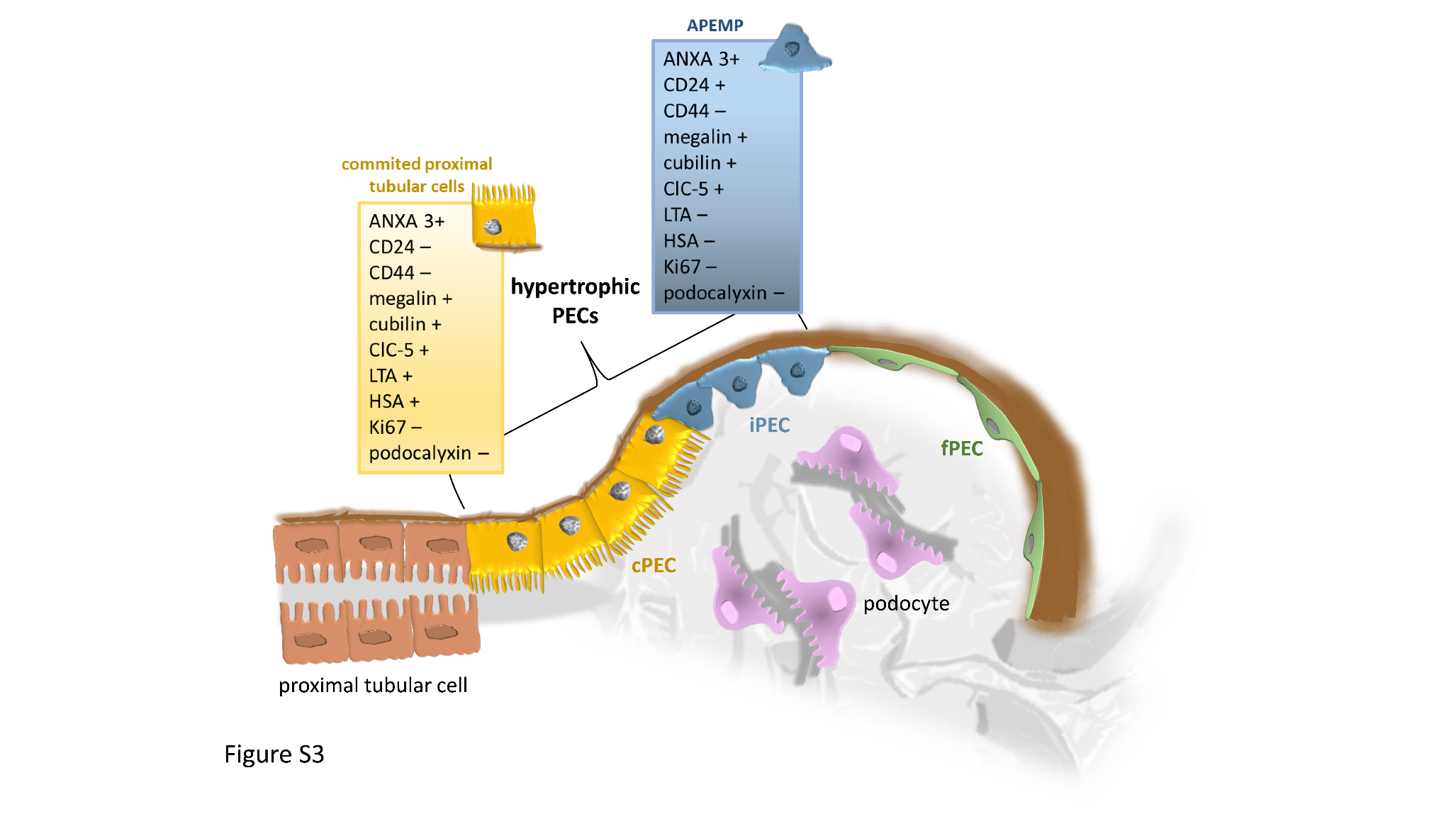


Intermediate PECs (APEMP) located at the tubular pole disclosing ANXA3^+^, CD24^+^, megalin^+^, cubilin^+^, ClC-5^+^, CD44^-^, LTA^-^, HSA^-^, Ki67^-^, Podocalyxin^-^ signals. Cuboid PECs (committed to proximal tubular cells) disclosing ANXA3^+^, CD24^-^, megalin^+^, cubilin^+^, ClC-5^+^, CD44^-^, LTA^+^, HSA^+^, Ki67^-^, Podocalyxin^-^ signals.
